# Discovery of a novel cytokine signature for the diagnosis of autism spectrum disorder in young Arab children in Qatar

**DOI:** 10.3389/fpsyt.2024.1333534

**Published:** 2024-02-13

**Authors:** Wared Nour-Eldine, Nimshitha Pavathuparambil Abdul Manaph, Samia M. Ltaief, Nazim Abdel Aati, Monaa Hussain Mansoori, Samya Al Abdulla, Abeer R. Al-Shammari

**Affiliations:** ^1^ Neurological Disorders Research Center, Qatar Biomedical Research Institute, Hamad Bin Khalifa University, Qatar Foundation, Doha, Qatar; ^2^ Child Development Center, Rumailah Hospital, Hamad Medical Corporation, Doha, Qatar; ^3^ Department of Operations, Primary Health Care Corporation, Doha, Qatar

**Keywords:** autism spectrum disorder, ASD, cytokines, biomarkers, luminex multiplex assay, logistic regression

## Abstract

**Background:**

Autism spectrum disorder (ASD) is a heterogeneous neurodevelopmental disorder characterized by impaired social interaction and communication and the occurrence of stereotyped and repetitive behaviors. Several studies have reported altered cytokine profiles in ASD and hence may serve as potential diagnostic biomarkers of the disorder. This study aims to identify diagnostic biomarkers for ASD in a well-defined study cohort in Qatar.

**Methods:**

We measured the protein levels of 45 cytokines in the plasma samples of age- and gender-matched children (2–4 years) with ASD (n = 100) and controls (n = 60) using a Luminex multiplex assay. We compared the differences in the levels of these cytokines between the two study groups and then fitted the significantly altered cytokines into a logistic regression model to examine their diagnostic potential for ASD.

**Results:**

We found elevated levels of IFN-γ, FGF-2, IL-1RA, and IL-13 and reduced levels of eotaxin, HGF, IL-1 alpha, IL-22, IL-9, MCP-1, SCF, SDF-1 alpha, VEGFA, and IP-10 in the plasma of children with ASD compared to controls. Furthermore, we observed that elevated levels of IFN-γ (odds ratio (OR) = 1.823; 95% (confidence interval) CI = 1.206, 2.755; p = 0.004) and FGF-2 (OR = 2.528; 95% CI = 1.457, 4.385; p < 0.001) were significantly associated with increased odds of ASD, whereas reduced levels of eotaxin (OR = 0.350; 95% CI = 0.160, 0.765; p = 0.008) and HGF (OR = 0.220; 95% CI = 0.070, 0.696; p = 0.010) were significantly associated with lower odds of ASD relative to controls. The combination of these four cytokines revealed an area under the curve (ROC-AUC) of 0.829 (95% CI = 0.767, 0.891; p < 0.001), which demonstrates the diagnostic accuracy of the four-cytokine signature.

**Conclusions:**

Our results identified a panel of cytokines that could discriminate between children with ASD and controls in Qatar. In addition, our findings support the predominance of a Th1 immune phenotype in ASD children and emphasize the need to validate these results in larger populations.

## Introduction

Autism spectrum disorder (ASD) is a heterogeneous neurodevelopmental disorder characterized by stereotyped, repetitive behaviors and impaired social communication skills. The worldwide prevalence of ASD is estimated to be 1 in 100 children with a global male-to-female ratio of 4.2 (1). Individuals with ASD generally show symptoms during the first three years of life, and the clinical severity of symptoms is largely variable among the affected individuals (2). Currently, there is no cure for ASD; however, studies have shown that early intervention programs are effective strategies in alleviating some of the severity symptoms (2). Nevertheless, there is often a long delay between referral and clinical evaluation and diagnosis of ASD by an expert team (3). This long delay in the clinical diagnosis of ASD impedes access to early intervention programs and hence may negatively impact the behavioral outcomes in the affected individuals. Accordingly, a major challenge remains to identify reliable biomarkers to support an early prediction of ASD, which could facilitate a timely access of individuals with ASD to the required support services (2).

Cytokines are signaling molecules that play key roles in the immune response. Current evidence supports a link between cytokines and ASD development (4, 5). Previous studies have reported abnormal levels of several cytokines in the peripheral blood of subjects with ASD (6). Remarkably, these dysregulated cytokines are associated with an increased severity of ASD symptoms (4, 5), suggesting a functional role of these cytokines in the disorder. For example, abnormal levels of several cytokines, such as interleukin (IL)-6, tumor necrosis factor (TNF)-α, and IL-1β, have been reported in the blood of subjects with ASD and are correlated with worse behavioral outcomes (7–11). Importantly, peripheral blood cytokines can cross the blood–brain barrier and signal to the brain, directly affecting brain function and behavior (12–15). Hence, circulating blood cytokines could present a promising source of potential biomarkers for ASD.

Several studies have investigated the utility of cytokines as biomarkers for ASD. However, the results of these studies have been inconsistent, which could be due to several factors. First, cytokines exist in a wide range and different studies have been selective in their measurement of certain types of cytokines compared to others (6). In addition, age, gender, and environmental exposures are other factors that may contribute to the discrepancies observed across studies. For example, most of the current studies analyzed cytokine levels in subjects with a wide age range of 3–12 years, and only a few studies have restricted the age range to 2–4 years to reduce the influence of age variations on the results (6). Finally, there may be other factors that could influence cytokine levels, such as medications or co-occurring conditions, which can further complicate the interpretation of the results. Adequate control of these variables is important for establishing more reliable cytokine biomarkers for ASD.

In this study, we sought to determine predictive biomarkers for early ASD diagnosis in a well-defined study cohort. Hence, we recruited a case–control study cohort of the Arab population in Qatar and used well-defined eligibility criteria to reduce the influence of confounding factors on the results. We utilized a Luminex multiplex assay to perform a simultaneous analysis of 45 cytokines in the plasma samples of the study cohort. We identified a unique panel of four cytokines that could predict ASD diagnosis in young children aged 2–4 years.

## Materials and methods

### Study participants

A total of 160 children were included in this study. We have employed a convenience sampling technique, in which the selection of the study participants was based on their accessibility and availability to take part in this study. Participants with ASD were recruited during the follow-up visits of the families to the Child Development Center in Rumailah Hospital of Hamad Medical Corporation (HMC) in Doha, Qatar and their matched control participants were recruited during the routine visits of families to the well-baby clinics or general clinics Al-Wajbah Health Center of Primary Health Care Corporation (PHCC) in Doha, Qatar.

All subjects were initially screened using a questionnaire to ensure that they met our study eligibility criteria. All enrolled subjects met the following criteria: age 2–4 years; Arabic ethnicity; born in Qatar; their mothers lived in Qatar during most of their pregnancy; enrolled subjects mostly lived in Qatar since birth; no immune conditions, such as autoimmune disease, asthma, allergy, and eczema; no neurological conditions, such as epilepsy; no suspected vision, hearing or walking problems; no other health problems, such as cardiovascular, lung, and kidney diseases; and not taking any medications and did not have any recent infection or vaccination at the time of study enrollment. No family history of ASD was another eligibility criterion for the control subjects. In addition, control subjects were screened using the validated Arabic version of the Social Communication Questionnaire (lifetime version) with a cutoff score < 12 for eligibility to rule out the risk of ASD in our control subjects (16). Control subjects were frequency matched to children with ASD based on age, gender, and nationality.

All participants with ASD had a confirmed clinical diagnosis of ASD by qualified professionals according to the Statistical Manual of Mental Disorders (DSM-5) and Autism Diagnostic Observation Schedule-second edition (ADOS-2) (17). Subjects with ADOS-2 scores < 7 were considered to have mild-to-moderate ASD, while subjects with ADOS-2 scores ≥ 7 were considered to have severe ASD. All families of the enrolled subjects completed a medical history questionnaire to collect their demographic data, as listed in **Table 1**.

**Table 1:** Demographic characteristics of the study population.

	Total (n = 160)	Control (n = 60)	ASD (n = 100)	p-value
**Age in years**	3.38 (2.99–3.73)	3.56 (2.98–3.81)	3.37 (2.99–3.70)	0.376
**Gender** Male Female	127 (79.4) 33 (20.6)	48 (80) 12 (20)	79 (79) 21 (21)	0.880
**Family history of ASD** Yes No	– –	NA NA	19 (19) 81 (81)	–
**Consanguinity** Yes No	48 (30) 112 (70)	21 (35) 39 (65)	27 (27) 73 (73)	0.285
**Method of reproduction** Natural Assisted (IVF)	153 (95.6) 7 (4.4)	59 (98.3) 1 (1.7)	94 (94) 6 (6)	0.257
**Maternal complications** **Yes** Diabetes Hypertension Asthma Allergy Multiple conditions (asthma, allergy, diabetes, and/or hypertension) **No**	62 (38.4) 39 (24.5) 5 (3.1) 2 (1.3) 3 (1.9) 12 (7.5) 98 (61.6)	19 (31.7) 14 (23.3) 0 (0) 1 (1.7) 0 (0) 4 (6.7) 41 (68.3)	43 (43) 25 (25.3) 5 (5.1) 1 (1.0) 3 (3.0) 8 (8.1) 57 (57)	0.154
**Pregnancy duration** < 9 months ≥ 9 months	10 (6.3) 150 (93.8)	2 (3.3) 58 (96.7)	8 (8) 92 (92)	0.323
**Maternal age at labor** Age in years < 35 years ≥ 35 years	30.00 (26.00–34.00) 126 (78.8) 34 (21.3)	29.50 (25.00–33.00) 51 (85) 9 (15)	30.00 (26.00–34.00) 75 (75) 25 (25)	0.289 0.134
**Type of delivery** Normal C-section Induced	101 (63.1) 52 (32.5) 7 (4.4)	42 (70) 17 (28.3) 1 (1.7)	59 (59) 35 (35) 6 (6)	0.242
**Postnatal complications** **Yes** Hypoxia Jaundice Hypoxia and Jaundice Others **No**	14 (8.8) 7 (4.4) 4 (2.5) 1 (0.6) 2 (1.3) 146 (91.3)	3 (5) 2 (3.3) 0 (0) 0 (0) 1 (1.7) 57 (95)	11 (11) 5 (5.0) 4 (4.0) 1 (1.0) 1 (1.0) 89 (89)	0.193
**Birth weight** Weight in kg < 2.5 kg ≥ 2.5 kg	3.00 (2.77–3.50) 23 (14.4) 137 (85.6)	3.00 (2.92–3.50) 6 (10) 54 (90)	3.00 (2.62–3.50) 17 (17) 83 (83)	0.484 0.222
**Nationality** Qatari Egyptian Syrian Yemeni Sudanese Jordanian Others	44 (27.5) 28 (17.5) 25 (15.6) 23 (14.4) 20 (12.5) 5 (3.1) 15 (9.4)	17 (28.3) 12 (20) 12 (20) 6 (10) 8 (13.3) 2 (3.3) 3 (5)	27 (27) 16 (16) 13 (13) 17 (17) 12 (12) 3 (3) 12 (12)	0.563

Data are presented as medians (lower – upper quartile) or n (%).

P-values were assessed by the Mann–Whitney U test for continuous variables and Pearson’s chi-square or Fisher’s exact test as appropriate for categorical variables.NA, Not Applicable.

### Plasma isolation and processing

Peripheral blood samples were extracted from study participants during the day or evening depending on the availability of the study participants and fasting was not requested for this study. The extracted blood samples were collected into EDTA-containing anticoagulant tubes at HMC or PHCC sites, transported, and processed in the research facility at QBRI within two hours of sample collection. Blood samples were slowly layered over Histopaque-1077 (cat #10771, Sigma–Aldrich) at an equal ratio and centrifuged at 400 *× g* for 30 min at a standard room temperature of ~ 21°C. After centrifugation, plasma samples were collected from the upper layer into new tubes and centrifuged at ~ 1800 *× g* for 15 min to remove cell debris. Plasma aliquots of 200 μl were stored at –80°C until further analysis.

### Cytokine measurements

Cytokine levels were measured in the plasma samples with a Luminex multiplex assay using a ProcartaPlex Convenience Panel 45-Plex kit (cat #EPXR450-12171-901, Thermo Fisher Scientific) according to the manufacturer’s instructions. The 45 cytokines analyzed were: BDNF, FGF-2, HGF, IL-1 alpha, IL-2, IL-6, IL-9, IL-13, IL-18, IL-23, IP-10, MIP-1 alpha, PDGF-BB, EGF, GM-CSF, IFN alpha, IL-1 beta, IL-4, IL-7, IL-10, IL-15, IL-21, IL-27, LIF, MIP-1 beta, PIGF-1, eotaxin, Gro-alpha, IFN-γ, IL-1RA, IL-5, IL-8, IL-12p70, IL-17A, IL-22, IL-31, MCP-1, NGF beta, RANTES, SCF, TNF beta, SDF-1 alpha, VEGF-A, TNF alpha, and VEGF-D. All samples and standards were measured in duplicate using the Bio-Plex 3D suspension array system (Bio-Rad Laboratories). Data analyses were performed using Bio-Plex Manager software (Bio-Rad Laboratories) according to the manufacturer’s instructions. Since logistic regression analysis cannot be conducted with missing values, analytes that fell below the limit of detection were assigned a value of half the minimum level of detection per each analyte, as reported in previous studies (18–23), and were included in the statistical analysis.

### Statistical analysis

Data were analyzed using the chi-square test for categorical variables and the Mann–Whitney U test for continuous variables. The Shapiro–Wilk normality test showed a nonparametric distribution of all data, and hence, the data are presented as median values with interquartile ranges. Data were considered statistically significant when the p-value < 0.05. Bonferroni correction was applied for the analysis of multiple comparisons.

For the binary logistic regression, data were natural log-transformed prior to the analysis. The outcome of interest was the diagnostic group of either ASD or control, and the predictors were only the significantly altered cytokines with adjusted p-values < 0.05. In the initial model, we adjusted for covariates of age, gender, sampling time (morning, afternoon, or evening), analysis batch number, and sample age (storage period from sample collection to cytokine analysis). We defined that a 10% change in the levels of the β-coefficient of the predictor determined which covariates to keep in the final model. Adjusted odd ratios (ORs) and 95% confidence intervals (CIs) were calculated to measure the association between each cytokine and ASD diagnosis relative to controls. Receiver operating characteristic (ROC) curves were constructed for the logistic regression model to assess the diagnostic accuracy of the selected cytokines in distinguishing between ASD and control cases. An area under the curve (ROC-AUC) was computed for each cytokine as well as the combination of cytokines using a nonparametric method. Data were analyzed using SPSS, version 26.0.

## Results

### Study population characteristics

As illustrated in **Table 1**, the study population showed matching demographic characteristics in terms of age, gender, and nationality. There was no significant difference in age between the ASD and control groups (p = 0.376, **Table 1**). The median age was 3.37 (2.99–3.70) years for the ASD group and 3.56 (2.98–3.81) years for the control group (**Table 1**). In addition, the study groups were gender matched at an almost 4:1 male-to-female ratio, 79/21 in ASD cases vs. 48/12 in the control group (p = 0.880, **Table 1**), which reflects the actual male bias in ASD. Moreover, the nationality distribution was not significantly different between the two study groups, with almost comparable percentages of Qatari (27 vs. 28.3%), Egyptian (16 vs. 20%), Syrian (13 vs. 20%), Yemeni (17 vs. 10%), Sudanese (12 vs. 13.3%), Jordanian (3 vs. 3.3%), and other citizens (12 vs. 5%) in the ASD vs. control groups (p = 0.563, **Table 1**).

Meanwhile, there were no differences between children with ASD and controls in terms of consanguinity, pregnancy duration, method of reproduction (natural or assisted), type of delivery (normal, c-section, or induced), maternal complications (diabetes, hypertension, asthma, or allergy), maternal age at delivery, birth weight, or postnatal complications (hypoxia or jaundice). Among the maternal complications recorded, it is worth noting the high yet comparable percentage of diabetes in the two study groups (25.3 vs. 23.3% in ASD vs. control group, **Table 1**), which is consistent with the previously reported high prevalence of gestational diabetes in the Qatari population (24). Finally, the ADOS-2 assessment scores of children with ASD are presented in **Table 2**.

**Table 2:** Clinical characteristics of children with ASD based on the ADOS-2 assessment.

	Mild-to-moderate ASD (n = 49)	Severe ASD (n = 51)
**Social affect**	9.00 (7.00–11.00)	19.00 (17.00–20.00)
**Restricted and repetitive behavior**	2.00 (1.00–2.50)	5.00 (3.00–6.00)
**Comparison score**	5.00 (4.00–6.00)	10.00 (8.00–10.00)

Data are presented as medians (lower – upper quartile).

### Altered plasma cytokine levels in ASD

We compared the plasma levels of 45 cytokines between the ASD and control groups, as summarized in **Table 3** and **Supplementary Table 1**. We found significantly elevated levels of FGF-2, IFN-γ, IL-13, and IL-1RA in addition to reduced levels of eotaxin, HGF, IL-1 alpha, IL-22, IL-9, IP-10, MCP-1, SCF, SDF-1 alpha, and VEGF-A in ASD compared to the control group (p < 0.05, **Table 3**). However, the results of only five cytokines (namely eotaxin, FGF-2, HGF, IFN-γ, and SDF-1 alpha) remained significant after Bonferroni correction for multiple comparisons (**Table 3**) and were thus selected for subsequent analysis. None of the plasma cytokines were notably altered between the mild-to-moderate and severe ASD subgroups, which suggest that these cytokines are not relevant markers for ASD severity classification (data not shown).

**Table 3:** Changes in the protein levels of several cytokines in the plasma of children with ASD compared to controls.

Cytokine	ASD (n = 100)	Control (n = 60)	p-value
**Eotaxin**	7.46 (5.12–10.83)	12.36 (7.69–16.88)	0.00087*
**FGF-2**	5.86 (5.30–11.44)	5.30 (1.81–5.86)	0.000219**
**HGF**	46.73 (37.66–61.92)	58.79 (46.67–73.09)	0.000347*
**IFN-γ**	15.84 (6.05–32.69)	6.24 (5.61–11.62)	0.000003***
**IL-13**	37.63 (21.56-49.56)	25.56 (17.60-45.12)	0.008
**IL-1a**	0.27 (0.26-0.44)	0.39 (0.35-0.47)	0.021
**IL-1RA**	97.62 (87.45-453.7)	87.45 (80.23-453.77)	0.019
**IL-22**	12.38 (11.01-12.74)	12.55 (12.38-12.74)	0.003
**IL-9**	3.73 (3.18-7.88)	4.60 (3.73-10.11)	0.023
**IP-10**	12.77 (10.28-16.16)	15.60 (12.28-19.78)	0.002
**MCP-1**	17.30 (11.39–33.62)	31.31 (18.91–44.97)	0.002
**SCF**	5.62 (4.17-7.77)	7.46 (4.73-9.57)	0.007
**SDF-1 alpha**	180.49 (68.26–403.56)	508.81 (248.55–814.41)	0.000005***
**VEGF-A**	27.81 (19.70-37.51)	33.92 (26.07-44.71)	0.007

Data are presented as medians (lower – upper quartile).

Mann–Whitney U test was used to assess statistical significance at p < 0.05.

Variables that remained statistically significant after Bonferroni correction by 45 variables are denoted with asterisks, *p < 0.05; **p < 0.01; ***p < 0.001.

### Logistic regression model revealed cytokine predictors of ASD

We performed binary logistic regression analysis to investigate the ability of the significantly altered cytokines to predict ASD diagnosis. In the initial model, we included the five significantly altered cytokines (eotaxin, FGF-2, HGF, IFN-γ, and SDF-1 alpha) and we adjusted for all the covariates in the model (namely age, gender, sampling time, analysis batch number, and sample age). Since none of these covariates were significant and removing them from the final model did not change the results, this indicates that these covariates were not confounding factors and hence were removed in the final model. We found that only four cytokines remained significant and hence were retained in the final model (**Table 4**). Elevated levels of IFN-γ (OR = 1.823; 95% CI = 1.206, 2.755; p = 0.004) and FGF-2 (OR = 2.528; 95% CI = 1.457, 4.385; p < 0.001) were associated with higher odds of having ASD whereas reduced levels of eotaxin (OR = 0.350; 95% CI = 0.160, 0.765; p = 0.008) and HGF (OR = 0.220; 95% CI = 0.070, 0.696; p = 0.010) were associated with lower odds of having ASD compared with the control group (**Table 4**).

**Table 4:** Binary logistic regression^a^ analysis of the altered cytokines revealed a list of cytokine predictors of ASD.

ASD vs. control			
Cytokine	Odds Ratio	95% CI	p-value
**Eotaxin**	0.350	(0.160, 0.765)	0.008
**FGF-2**	2.528	(1.457, 4.385)	< 0.001
**HGF**	0.220	(0.070, 0.696)	0.010
**IFN-γ**	1.823	(1.206, 2.755)	0.004

a Adjusted for age, gender, sampling time, sample age, and analysis batch number in the initial model, then removed these covariates in the final model as they were not confounding factors.

### Diagnostic accuracy of the identified ASD biomarkers

**Figure 1:**
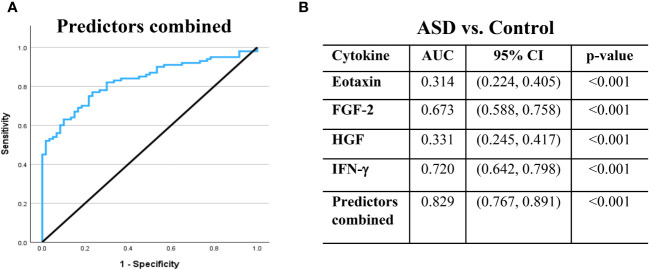
Receiver operating characteristic (ROC) curves demonstrate the ability of the selected cytokines to differentiate between ASD and control cases. **(A)** ROC curve is illustrated for the combination of the four predictors. **(B)** The area under the ROC curve (AUC), 95% confidence interval (CI) and p-value are displayed in table for the individual and combined predictors. AUC values above 0.5 suggest a positive association with ASD, whereas AUC values below 0.5 suggest a negative association. The black line is the reference line.

We fitted ROC curves to examine the performance of the four significant cytokines that remained in the final logistic regression model (**Figure 1**). ROC curve analysis showed that each of the four cytokines demonstrated significant performance (p < 0.001, **Figure 1**). Specifically, IFN-γ showed an area under the curve (AUC) = 0.720 with 95% CI = (0.642, 0.798), FGF-2 (AUC = 0.673; 95% CI = 0.588, 0.758), eotaxin (AUC = 0.314; 95% CI = 0.224, 0.405), and HGF (AUC = 0.331; 95% CI = 0.245, 0.417). An AUC below 0.5 for eotaxin and HGF indicates lower levels of these cytokines in the ASD group than in the control group. Remarkably, ROC curve analysis demonstrated that the combination of the four cytokines (IFN-γ, FGF-2, eotaxin, and HGF) showed the best diagnostic accuracy for ASD with AUC = 0.829 and 95% CI = 0.767, 0.891 (p < 0.001, **Figure 1**).

## Discussion

This study aimed to identify cytokine biomarkers for early ASD diagnosis. For this purpose, we recruited a well-defined cohort of ASD and control subjects in Qatar with matching age, gender, ethnicity, and clinical characteristics. We analyzed cytokine levels in the plasma samples of our cohort to determine their potential utility as early diagnostic biomarkers for ASD. We found that 14 cytokines were significantly altered in ASD individuals compared to controls. However, only five cytokines remained significant after correcting for multiple comparisons. We then subjected these five cytokines to a logistic regression analysis to examine their ability to differentiate between ASD and control cases. Remarkably, we found that a combination of four cytokines, namely eotaxin, FGF-2, HGF, and IFN-γ, represent the best predictors of ASD with an overall accuracy exceeding 80%. To the best of our knowledge, this is the first exploratory study to provide a cytokine-based model for predicting ASD diagnosis in young Arab children in the Qatari population. We propose the potential use of this four-cytokine panel in early ASD diagnosis, which remains to be validated in independent studies.

We observed elevated levels of IFN-γ and reduced levels of eotaxin in the plasma samples in ASD. Previous studies support our finding of increased plasma levels of IFN-γ in ASD (25–27). Furthermore, elevated IFN-γ levels were previously reported in the mid-gestational serum of mothers to children ultimately diagnosed with ASD (28) as well as in the frontal cerebral cortex of individuals with ASD (29). Remarkably, dysregulated IFN-γ signaling contributes to increased neurite outgrowth (30), abnormal neuronal connectivity and social dysfunction (31), which together reflect a typical ASD phenotype. In contrast, we found reduced levels of eotaxin in ASD, which contradicts previous studies that reported increased levels of eotaxin in ASD (8, 32). In response to innate immune activation, the proinflammatory cytokine IFN-γ maintains T helper 1 (Th1) lineage commitment and directs Th1 immune responses while inhibiting differentiation toward other Th cell subsets, including Th2 cells (33). Meanwhile, eotaxin is a chemotactic cytokine that selectively recruits eosinophils to inflammatory sites and orchestrates Th2 effector mechanisms (34). Our results of reduced levels of eotaxin and increased levels of IFN-γ suggest a dampened Th2 transcriptional program and a shift toward the Th1 phenotype in ASD. This finding supports our recently published systematic review that proposes a preferential polarization toward the Th1 phenotype in ASD (6).

On the other hand, we found elevated FGF-2 levels and reduced HGF levels in ASD. FGF-2, also known as basic fibroblast growth factor (bFGF), is a multifunctional growth factor that plays critical roles in neurodevelopment and synaptic plasticity and modulates neuroinflammatory responses (35). A previous study reported decreased serum levels of FGF-2 in individuals with ASD (36), with opposing results to our findings. However, studies on other psychiatric disorders reported increased serum levels of FGF-2 in individuals with schizophrenia (37). In addition, increased expression of FGF-2 receptor (FGFR1) was found in the hippocampus of postmortem brains of patients with schizophrenia and major depression (38). In contrast, HGF is a key factor that prevents neuronal death and promotes survival through pro-angiogenic and immunomodulatory mechanisms. Ablation of HGF or its receptor, MET, affects synaptic plasticity in the brain (39). Previous studies reported decreased serum levels of HGF in ASD (40, 41), which supports our results. Furthermore, reduced MET protein levels were found in the postmortem cerebral cortex of individuals with ASD (42). Interestingly, decreased MET gene expression and decreased HGF protein levels were associated with ASD and co-occurring gastrointestinal conditions (41, 43, 44). Taken together, dysregulated levels of FGF-2 and HGF in ASD may contribute to disrupted immune and neuronal functions associated with ASD.

Several studies attempted to identify reliable cytokine biomarkers for ASD; however, the outcomes remain largely inconsistent, which could be due to differences in cohort characteristics and sample analysis approaches across different studies. Although current studies used standard methods for cytokine detection, such as ELISA, cytokine multiplex, or flow cytometry, significant differences might still exist regarding sample handling and processing methods, the analysis platforms used, and the sensitivity and specificity of the antibodies used across different studies (6). In addition, current studies are widely variable in the clinical characteristics of study cohorts, such as age, gender, ethnicity, and co-occurring conditions, which could contribute to the variability in the results across studies. Most of the current studies included subjects in the age range between 2 and 12 years (6). However, it is important to emphasize that the immune system undergoes dynamic changes during the developmental period from early childhood to adolescence (45), and hence some variability might be introduced when comparing the results of studies that included subjects with different age ranges. Furthermore, many of the current studies lacked details of the clinical features and co-occurring conditions of the study participants (6), which could further contribute to result variability across studies. In this study, we restricted the age range of subjects to 2–4 years to minimize the effect of age variability on the results and to identify reliable biomarkers for the early diagnosis of ASD at a timepoint during which most ASD symptoms appear. In addition, we focused on subjects without known co-occurring clinical conditions to minimize possible confounding effects of these conditions on the results. Finally, we focused on a specific ethnicity of the Arab population to reduce any influence of genetic variability on the results, and we ensured that the study population represented a 4 to 1 male-to-female ratio to reflect the typical gender distribution in ASD.

In conclusion, we report dysregulated levels of several cytokines in the plasma samples of young children with ASD. We identified a novel cytokine panel of IFN-γ, eotaxin, HGF, and FGF-2 as the best predictive markers of early ASD diagnosis with an overall diagnostic accuracy exceeding 80%. Additionally, our data imply the predominance of the Th1-like immune phenotype in ASD children. These findings require further validation in independent cohorts. We emphasize the importance of recruiting a well-defined study population in future studies on ASD to improve the accuracy and reproducibility of the results. In addition, there is a need for more longitudinal analyses in future studies to correlate the time-dependent changes in cytokine production with the clinical and behavioral phenotypes in ASD.

## Data availability statement

The raw data supporting the conclusions of this article will be made available by the authors, without undue reservation.

## Ethics statement

The studies involving humans were approved by the Institutional Review Board (IRB) Ethics Committee of HMC (approval number: MRC-02-18-116) for the recruitment of subjects with ASD and the PHCC (approval number: 2020/06/064) for the recruitment of control subjects. The studies were conducted in accordance with the local legislation and institutional requirements. Written informed consent for participation in this study was provided by the participants’ legal guardians/next of kin.

## Author contributions

WN-E: Writing – original draft, Writing – review & editing, Data curation, Formal analysis, Investigation, Methodology, Visualization. NM: Investigation, Methodology, Writing – review & editing. SL: Writing – review & editing, Data curation, Project administration, Resources. NA: Writing – review & editing, Supervision. MM: Supervision, Writing – review & editing. SA: Supervision, Writing – review & editing. AA-S: Supervision, Writing – review & editing, Conceptualization, Funding acquisition, Project administration, Writing – original draft, Investigation.

## Supplementary material

Supplementary Table 1 Comparison of the plasma cytokine levels in children with ASD and the control group. Data are presented as percentages or medians (lower – upper quartile) with p-values of the Mann—Whitney U test. Bold numerals indicate statistical significance (p-value < 0.05).

## References

[1] Zeidan J Fombonne E Scorah J Ibrahim A Durkin MS Saxena S. Global prevalence of autism: A systematic review update. Autism Res (2022) 15(5):778–90. doi: 10.1002/aur.2696 PMC9310578 35238171

[2] Lord C Elsabbagh M Baird G Veenstra-Vanderweele J. Autism spectrum disorder. Lancet (2018) 392(10146):508–20. doi: 10.1016/S0140-6736(18)31129-2 PMC7398158 30078460

[3] Lord C Charman T Havdahl A Carbone P Anagnostou E Boyd B. The Lancet Commission on the future of care and clinical research in autism. Lancet (2022) 399(10321):271–334. doi: 10.1016/S0140-6736(21)01541-5 34883054

[4] Gladysz D Krzywdzinska A Hozyasz KK. Immune abnormalities in autism spectrum disorder-could they hold promise for causative treatment? Mol Neurobiol (2018) 55(8):6387–435. doi: 10.1007/s12035-017-0822-x PMC6061181 29307081

[5] Onore C Careaga M Ashwood P. The role of immune dysfunction in the pathophysiology of autism. Brain Behav Immun (2012) 26(3):383–92. doi: 10.1016/j.bbi.2011.08.007 PMC3418145 21906670

[6] Nour-Eldine W Ltaief SM Abdul Manaph NP Al-Shammari AR. In search of immune cellular sources of abnormal cytokines in the blood in autism spectrum disorder: A systematic review of case-control studies. Front Immunol (2022) 13:950275. doi: 10.3389/fimmu.2022.950275 36268027 PMC9578337

[7] Ashwood P Krakowiak P Hertz-Picciotto I Hansen R Pessah I Van de Water J. Elevated plasma cytokines in autism spectrum disorders provide evidence of immune dysfunction and are associated with impaired behavioral outcome. Brain Behavior Immun (2011) 25(1):40–5. doi: 10.1016/j.bbi.2010.08.003 PMC2991432 20705131

[8] Ashwood P Krakowiak P Hertz-Picciotto I Hansen R Pessah IN Van de Water J. Associations of impaired behaviors with elevated plasma chemokines in autism spectrum disorders. J Neuroimmunology (2011) 232(1-2):196–9. doi: 10.1016/j.jneuroim.2010.10.025 PMC3053074 21095018

[9] Enstrom AM Onore CE Van de Water JA Ashwood P. Differential monocyte responses to TLR ligands in children with autism spectrum disorders. Brain Behavior Immun (2010) 24(1):64–71. doi: 10.1016/j.bbi.2009.08.001 PMC3014091 19666104

[10] Saad K Abdallah AM Abdel-Rahman AA Al-Atram AA Abdel-Raheem YF Gad EF. Polymorphism of interleukin-1β and interleukin-1 receptor antagonist genes in children with autism spectrum disorders. Prog Neuropsychopharmacol Biol Psychiatry (2020) 103:109999. doi: 10.1016/j.pnpbp.2020.109999 32526258

[11] Xie J Huang L Li X Li H Zhou Y Zhu H. Immunological cytokine profiling identifies TNF-α as a key molecule dysregulated in autistic children. Oncotarget (2017) 8(47):82390–8. doi: 10.18632/oncotarget.19326 PMC5669898 29137272

[12] Estes ML McAllister AK. Immune mediators in the brain and peripheral tissues in autism spectrum disorder. Nat Rev Neurosci (2015) 16(8):469–86. doi: 10.1038/nrn3978 PMC5650494 26189694

[13] Yarlagadda A Alfson E Clayton AH. The blood brain barrier and the role of cytokines in neuropsychiatry. Psychiatry (Edgmont) (2009) 6(11):18–22. PMC2801483 20049146

[14] Gottfried C Bambini-Junior V Francis F Riesgo R Savino W. The impact of neuroimmune alterations in autism spectrum disorder. Front Psychiatry (2015) 6:121. doi: 10.3389/fpsyt.2015.00121 26441683 PMC4563148

[15] Siniscalco D Schultz S Brigida AL Antonucci N. Inflammation and neuro-immune dysregulations in autism spectrum disorders. Pharm (Basel) (2018) 11(2):56. doi: 10.3390/ph11020056 PMC6027314 29867038

[16] Aldosari M Fombonne E Aldhalaan H Ouda M Elhag S Alshammari H. Validation of the Arabic version of the social communication questionnaire. Autism (2019) 23(7):1655–62. doi: 10.1177/1362361318816065 PMC6728746 30606031

[17] Lord C Rutter M DiLavore P Risi S Gotham K Bishop S. Autism diagnostic observation schedule–2nd edition (ADOS-2). Los Angeles, CA: Western Psychological Corporation (2012). p. 284.

[18] Ashwood P Krakowiak P Hertz-Picciotto I Hansen R Pessah IN Van de Water J. Altered T cell responses in children with autism. Brain Behav Immun (2011) 25(5):840–9. doi: 10.1016/j.bbi.2010.09.002 PMC3039713 20833247

[19] Zerbo O Yoshida C Grether JK Van de Water J Ashwood P Delorenze GN. Neonatal cytokines and chemokines and risk of Autism Spectrum Disorder: the Early Markers for Autism (EMA) study: a case-control study. J Neuroinflamm (2014) 11:113. doi: 10.1186/1742-2094-11-113 PMC4080514 24951035

[20] Rose DR Yang H Serena G Sturgeon C Ma B Careaga M. Differential immune responses and microbiota profiles in children with autism spectrum disorders and co-morbid gastrointestinal symptoms. Brain Behav Immun (2018) 70:354–68. doi: 10.1016/j.bbi.2018.03.025 PMC5953830 29571898

[21] Heuer LS Croen LA Jones KL Yoshida CK Hansen RL Yolken R. An exploratory examination of neonatal cytokines and chemokines as predictors of autism risk: the early markers for autism study. Biol Psychiatry (2019) 86(4):255–64. doi: 10.1016/j.biopsych.2019.04.037 PMC6677631 31279535

[22] Manzardo AM Henkhaus R Dhillon S Butler MG. Plasma cytokine levels in children with autistic disorder and unrelated siblings. Int J Dev Neurosci (2012) 30(2):121–7. doi: 10.1016/j.ijdevneu.2011.12.003 PMC6675569 22197967

[23] Shen Y Li Y Shi L Liu M Wu R Xia K. Autism spectrum disorder and severe social impairment associated with elevated plasma interleukin-8. Pediatr Res (2021) 89(3):591–7. doi: 10.1038/s41390-020-0910-x 32330928

[24] Bashir M M EA-R Aboulfotouh M Eltaher F Omar K Babarinsa I. Prevalence of newly detected diabetes in pregnancy in Qatar, using universal screening. PloS One (2018) 13(8):e0201247. doi: 10.1371/journal.pone.0201247 30074993 PMC6075760

[25] El-Ansary A Al-Ayadhi L. GABAergic/glutamatergic imbalance relative to excessive neuroinflammation in autism spectrum disorders. J Neuroinflamm (2014) 11:189. doi: 10.1186/s12974-014-0189-0 PMC4243332 25407263

[26] Tostes MH Teixeira HC Gattaz WF Brandão MA Raposo NR. Altered neurotrophin, neuropeptide, cytokines and nitric oxide levels in autism. Pharmacopsychiatry (2012) 45(6):241–3. doi: 10.1055/s-0032-1301914 22426848

[27] El-Ansary A Al-Ayadhi L. Neuroinflammation in autism spectrum disorders. J Neuroinflamm (2012) 9:265. doi: 10.1186/1742-2094-9-265 PMC3549857 23231720

[28] Goines PE Croen LA Braunschweig D Yoshida CK Grether J Hansen R. Increased midgestational IFN-γ, IL-4 and IL-5 in women bearing a child with autism: A case-control study. Mol Autism (2011) 2(1):13. doi: 10.1186/2040-2392-2-13 21810230 PMC3170586

[29] Li X Chauhan A Sheikh AM Patil S Chauhan V Li XM. Elevated immune response in the brain of autistic patients. J Neuroimmunol (2009) 207(1-2):111–6. doi: 10.1016/j.jneuroim.2008.12.002 PMC2770268 19157572

[30] Warre-Cornish K Perfect L Nagy R Duarte RRR Reid MJ Raval P. Interferon-γ signaling in human iPSC-derived neurons recapitulates neurodevelopmental disorder phenotypes. Sci Adv (2020) 6(34):eaay9506. doi: 10.1126/sciadv.aay9506 32875100 PMC7438100

[31] Filiano AJ Xu Y Tustison NJ Marsh RL Baker W Smirnov I. Unexpected role of interferon-γ in regulating neuronal connectivity and social behaviour. Nature (2016) 535(7612):425–9. doi: 10.1038/nature18626 PMC4961620 27409813

[32] Hu CC Xu X Xiong GL Xu Q Zhou BR Li CY. Alterations in plasma cytokine levels in chinese children with autism spectrum disorder. Autism Res (2018) 11(7):989–99. doi: 10.1002/aur.1940 29522267

[33] Butcher MJ Zhu J. Recent advances in understanding the Th1/Th2 effector choice. Fac Rev (2021) 10:30. doi: 10.12703/r/10-30 33817699 PMC8009194

[34] Teixeira AL Gama CS Rocha NP Teixeira MM. Revisiting the role of eotaxin-1/CCL11 in psychiatric disorders. Front Psychiatry (2018) 9:241. doi: 10.3389/fpsyt.2018.00241 29962972 PMC6010544

[35] Woodbury ME Ikezu T. Fibroblast growth factor-2 signaling in neurogenesis and neurodegeneration. J Neuroimmune Pharmacol (2014) 9(2):92–101. doi: 10.1007/s11481-013-9501-5 24057103 PMC4109802

[36] Esnafoglu E Ayyıldız SN. Decreased levels of serum fibroblast growth factor-2 in children with autism spectrum disorder. Psychiatry Res (2017) 257:79–83. doi: 10.1016/j.psychres.2017.07.028 28734240

[37] Hashimoto K Shimizu E Komatsu N Nakazato M Okamura N Watanabe H. Increased levels of serum basic fibroblast growth factor in schizophrenia. Psychiatry Res (2003) 120(3):211–8. doi: 10.1016/S0165-1781(03)00186-0 14561432

[38] Gaughran F Payne J Sedgwick PM Cotter D Berry M. Hippocampal FGF-2 and FGFR1 mRNA expression in major depression, schizophrenia and bipolar disorder. Brain Res Bull (2006) 70(3):221–7. doi: 10.1016/j.brainresbull.2006.04.008 16861106

[39] Desole C Gallo S Vitacolonna A Montarolo F Bertolotto A Vivien D. HGF and MET: from brain development to neurological disorders. Front Cell Dev Biol (2021) 9:683609. doi: 10.3389/fcell.2021.683609 34179015 PMC8220160

[40] Sugihara G Hashimoto K Iwata Y Nakamura K Tsujii M Tsuchiya KJ. Decreased serum levels of hepatocyte growth factor in male adults with high-functioning autism. Prog Neuropsychopharmacol Biol Psychiatry (2007) 31(2):412–5. doi: 10.1016/j.pnpbp.2006.10.010 17157424

[41] Russo AJ Krigsman A Jepson B Wakefield A. Decreased serum hepatocyte growth factor (HGF) in autistic children with severe gastrointestinal disease. biomark Insights (2009) 4:181–90. doi: 10.4137/BMI.S3656 PMC2796865 20029653

[42] Campbell DB D'Oronzio R Garbett K Ebert PJ Mirnics K Levitt P. Disruption of cerebral cortex MET signaling in autism spectrum disorder. Ann Neurol (2007) 62(3):243–50. doi: 10.1002/ana.21180 17696172

[43] Campbell DB Sutcliffe JS Ebert PJ Militerni R Bravaccio C Trillo S. A genetic variant that disrupts MET transcription is associated with autism. Proc Natl Acad Sci USA (2006) 103(45):16834–9. doi: 10.1073/pnas.0605296103 PMC1838551 17053076

[44] Campbell DB Buie TM Winter H Bauman M Sutcliffe JS Perrin JM. Distinct genetic risk based on association of MET in families with co-occurring autism and gastrointestinal conditions. Pediatrics (2009) 123(3):1018–24. doi: 10.1542/peds.2008-0819 19255034

[45] Simon AK Hollander GA McMichael A. Evolution of the immune system in humans from infancy to old age. Proc Biol Sci (2015) 282(1821):20143085. doi: 10.1098/rspb.2014.3085 26702035 PMC4707740

