# Supplementary material for: Discovery of a novel cytokine signature for the diagnosis of autism spectrum disorder in young Arab children in Qatar
Source: Front Psychiatry. 2024 Feb 13;15:1333534. doi: 10.3389/fpsyt.2024.1333534 (PMC10896998; doi:10.3389/fpsyt.2024.1333534)
Supplement: Supplementary Table 1 — Comparison of the plasma cytokine levels in children with ASD and the control group. Data are presented as percentages or medians (lower – upper quartile) with p-values of the Mann—Whitney U test. Bold numerals indicate statistical significance (p-value < 0.05). [file Table_1.docx]

**Supplementary Material**

**Supplementary Table 1.** Comparison of the plasma cytokine levels in children with ASD and the control group.

| Cytokine | % Samples below the limit of detection | Control  (n = 60) | ASD  (n = 100) | p-value |
| --- | --- | --- | --- | --- |
| BDNF | 1% | 20.86  (11.44–42.49) | 25.32  (13.65–36.86) | 0.913 |
| EGF | 23% | 9.37  (1.05–18.78) | 12.69  (5.99–21.52) | 0.175 |
| Eotaxin | 0% | 12.36  (7.69–16.88) | 7.46  (5.12–10.83) | **0.000087** |
| FGF-2 | 74% | 5.30  (1.81–5.86) | 5.86  (5.30–11.44) | **0.000219** |
| GM-CSF | 4% | 112.65  (58.93–158.69) | 84.92  (48.07–144.63) | 0.108 |
| Gro-alpha | 43% | 1.42  (1.16–4.10) | 2.61  (1.20–5.44) | 0.062 |
| HGF | 0% | 58.79  (46.67–73.09) | 46.73  (37.66–61.92) | **0.000347** |
| IFN-alpha | 17% | 1.14  (0.66–1.48) | 0.93  (0.52–1.47) | 0.252 |
| IFN-γ | 43% | 6.24  (5.61–11.62) | 15.84  (6.05–32.69) | **0.000003** |
| IL-10 | 19% | 5.81  (1.33–9.89) | 5.30  (2.00–9.35) | 0.779 |
| IL-12p70 | 42% | 3.38  (1.81–4.70) | 3.30  (2.01–4.58) | 0.735 |
| IL-13 | 10% | 25.56  (17.60–45.12) | 37.63  (21.56–49.56) | **0.008** |
| IL-15 | 2% | 39.02  (22.28–68.58) | 28.54  (20.07–53.44) | 0.149 |
| IL-17A | 6% | 27.36  (6.71–45.53) | 15.54  (7.41–31.55) | 0.080 |
| IL-18 | 0% | 72.53  (49.56–106.78) | 79.01  (55.76–101.43) | 0.448 |
| IL-1a | 64% | 0.39  (0.35–0.47) | 0.27  (0.26–0.44) | **0.021** |
| IL-1 beta | 3% | 15.92  (9.32–27.34) | 18.51  (12.50–27.30) | 0.271 |
| IL-1RA | 88% | 87.45  (80.23–453.77) | 97.62  (87.45–453.77) | **0.019** |
| IL-2 | 3% | 31.99  (16.92–41.18) | 30.06  (19.59–40.81) | 0.820 |
| IL-21 | 63% | 8.20  (7.61–28.38) | 7.61  (5.62–36.33) | 0.167 |
| IL-22 | 76% | 12.55  (12.38–12.74) | 12.38  (11.01–12.74) | **0.003** |
| IL-23 | 55% | 29.00  (8.54–43.57) | 29.00  (21.99–71.79) | 0.126 |
| IL-27 | 9% | 134.52  (69.75–273.73) | 162.37  (82.24–256.51) | 0.654 |
| IL-31 | 56% | 5.89  (5.64–19.26) | 6.50  (5.39–53.1) | 0.811 |
| IL-4 | 40% | 10.67  (6.45–16.30) | 12.63  (6.36–20.60) | 0.166 |
| IL-5 | 7% | 53.30  (31.59–72.81) | 47.54  (30.08–68.16) | 0.386 |
| IL-6 | 4% | 65.58  (36.35–122.70) | 71.51  (39.34–117.27) | 0.457 |
| IL-7 | 1% | 5.75  (3.55–8.93) | 6.09  (3.57–10.60) | 0.648 |
| IL-8 | 42% | 2.74  (1.51–6.40) | 3.02  (0.98–7.02) | 0.446 |
| IL-9 | 72% | 4.60  (3.73–10.11) | 3.73  (3.18–7.88) | **0.023** |
| IP-10 | 0% | 15.60  (12.28–19.78) | 12.77  (10.28–16.16) | **0.002** |
| LIF | 3% | 8.29  (5.26–10.61) | 6.77  (4.79–10.12) | 0.248 |
| MCP-1 | 0% | 31.31  (18.91–44.97) | 17.30  (11.39–33.62) | **0.002** |
| MIP 1 alpha | 43% | 7.39  (0.92–27.06) | 3.90  (0.92–21.57) | 0.689 |
| MIP 1 beta | 48% | 3.20  (2.83–16.20) | 7.60  (2.83–16.20) | 0.484 |
| PDGF BB | 0% | 165.00  (84.60–353.25) | 154.42  (92.22–336.83) | 0.805 |
| PIGF-1 | 44% | 1.02  (0.95–13.33) | 8.15  (0.96–16.23) | 0.383 |
| RANTES | 0% | 88.91  (67.27–115.05) | 78.86  (56.43–108.95) | 0.129 |
| SCF | 0% | 7.46  (4.73–9.57) | 5.62  (4.17–7.77) | **0.007** |
| SDF-1 alpha | 16% | 508.81  (248.55–814.41) | 180.49  (68.26–403.56) | **0.00050** |
| TNF-alpha | 11% | 14.25  (10.82–21.49) | 16.74  (10.49–23.12) | 0.320 |
| TNF-beta | 69% | 2.69  (2.62–12.13) | 2.69  (2.43–21.42) | 0.397 |
| VEGF-A | 0.6% | 33.92  (26.07–44.71) | 27.81  (19.70–37.51) | **0.007** |
| VEGF-D | 43% | 0.98  (0.77–3.73) | 1.88  (0.88–5.04) | 0.123 |
| bNGF | 11% | 2.08  (0.70–3.72) | 2.00  (0.91–4.01) | 0.239 |

Data are presented as percentages or medians (lower ­– upper quartile) with p-values of the Mann–Whitney U test.

Bold numerals indicate statistical significance (p-value < 0.05).
